# Changes in the hydro-sedimentary balance: Impacts of the use of a borrow pit in a low-order stream

**DOI:** 10.1371/journal.pone.0255432

**Published:** 2021-08-09

**Authors:** Cláudio Tavares, Eneida M. Eskinazi-Sant’Anna, Yuri A. Figueiredo, Hernani A. Almeida, Mariangela G. P. Leite

**Affiliations:** 1 Graduate Program in Crustal Evolution and Natural Resources, Federal University of Ouro Preto, Minas Gerais, Brazil; 2 Department of Biodiversity, Evolution and Environment, Federal University of Ouro Preto, Minas Gerais, Brazil; Jinan University, CHINA

## Abstract

Construction of dams for hydroelectric power requires significant quantities of soil and rock, which are often extracted in borrow pits from adjacent regions. Although the effects of dams on stream processes has received significant attention, the effects of borrow pits has not. The main objective of this study was to analyze the geomorphological and sedimentological aspects of two second-order streams, one of which was directly affected by the borrow pit located upstream of its source (Pedra Branca stream). Flow rates were measured and cross-sections of 600m stretches in both streams were monitored over a hydrological year. At the same time, sediments from the bed of the channels and soils on their banks had their physical and chemical characteristics evaluated. Streams sediments differed in their chemical and organic matter composition. The mean particle size of the sediment particles was different between the reference and degraded streams. The water flow was very similar to both streams, only varying along the seasonal seasons. However, the fluvial channels presented great geomorphological differentiation, mainly downstream, due to the location of the Pedra Branca stream and its proximity to the borrow pit. Despite the great importance for the production of clean electric energy, the construction of hydroelectric plants promotes persistent impacts that affect structural and functional aspects of the adjacent aquatic habitats. Borrow pits used for the construction of projects become large sources of sediment for aquatic environments, affecting the drainage network of the hydrographic basin and the balance of river erosion, transport and deposition processes. The results show the need to review the intervention protocols in borrow pits and the environmental legislation that regulates their rehabilitation.

## Introduction

The 20th century was marked by the construction of large hydroelectric plants in response to the demand for cheaper and cleaner energy [1] and hundreds of reservoirs were built around the world. However, the construction of large hydroelectric plants is among the most controversial development projects [2]. Brazil is the country that most uses renewable sources in the production of electric energy, and almost 83% of its energy matrix coming from renewable sources [3]. Out of the total electric energy produced in the country, more than 63% is hydroelectric energy, with 1375 plants spread across the national territory [3,4]. Despite being a source of renewable electricity and the most cost-effective electricity, especially for large urban centers, they are responsible for a wide range of environmental impacts [5–7].

The negative impacts on water resources resulting from the construction of a hydroelectric plant are well studied and relatively well known [8,9]. However, damage to the environment is not concentrated only in the region directly affected by the reservoir. For the implementation of this energy matrix, it is necessary to build large dams, whose infrastructure construction involves the removal of vegetation cover from large areas to obtain stones, gravel, sand and soil [2,10]. The areas that provide these materials are commonly known as quarries and borrow pits, normally located in the region surrounding the project area [11–13]. In the case of borrow pits for the removal of clays, in addition to the elimination of vegetation, part or all of the surface horizons of the soil are also removed, sometimes exposing the C horizon [12,14].

In hydrographic basins where vegetation has been suppressed and the surface layers of soil have been removed, significant changes in the water cycle occur, with a reduction in infiltration rates and increased runoff [15]. In many cases the increase in laminar flow, in conjunction with the mechanisms of impact of intense rain drops on exposed soil, is responsible for the disaggregation of a large part of the material that will be carried by surface runoff [16]. In extreme cases, severe erosion processes can occur, with the establishment of ravines, caves and even gullies [10]. It is precisely the anthropized areas of tropical regions that have the highest volumes of runoff and, consequently, the highest rates of soil loss through erosion [17].

Streams and rivers, like all geomorphological systems, are in a metastable dynamic balance [18]. This balance is defined, roughly, by the relationship between transport capacity and the material available for this transport [16,19]. Its geomorphologies are a combined response between long-term (related to geological processes) and short-term evolutionary processes (responses to changes in hydrology and sediment transport, [20]. The increase in the sediment load of rivers and streams, promoted directly or indirectly by anthropic actions, leads to a hydro-sedimentary imbalance, resulting in the deposition of excess sediment load and silting [7,21]. Silting causes major impacts on lotic systems, affecting some of the main aspects of its geomorphology, such as the shape and complexity of the channel, the flow connectivity and the dynamism of its deposits [7,22]. Consequently, it is observed a homogenization of the bed, the loss of microhabitats and impacts on aquatic biodiversity [23,24].

This paper aims to describe the impacts of the borrow pits on hydrographic basins under their influence, an aspect that has not yet been explored in the assessment of the impacts of the construction of reservoirs in Brazil. Almost always, the effects caused by the borrow pits are neglected. The lack of cause and effect studies in the borrow pits promotes the lack of better defined protocols to mitigate the impacts that are caused by them. In this context, the main goal of the present study was to evaluate the impacts on the hydro-sedimentary balance of the Pedra Branca stream, whose source region was used as a soil borrow pit for the construction of the Emborcação reservoir (Catalão, GO). The following predictions were tested: (i) the erosive processes, strongly enhanced by the stripping and removal of soil from the borrow pit, promoted changes in the liquid discharge and in the sediments of the Pedra Branca stream; (ii) variations in the dynamic balance of the impacted stream, especially in the rainy season, lead to changes in its geomorphology; (iii) in response to hydro-sedimentological changes, there will be a change in the physical and chemical composition of the river sediments.

## Materials and methods

### Study area

The study site corresponds to a borrow pit from which soil was removed for the construction of the Emborcação Hydroelectric Plant (Teodomiro Santiago Reservoir). The degraded area (DA) resulting from the borrow pit covers an area of approximately 220 ha, and is located in the district of Pedra Branca, Catalão, GO (18° 26’0.00 "S and 47° 56’57.41" W). The DA includes areas with exposed soil (without any type of vegetation cover), with remnants of native forest (gallery forests and dense dry tropical forests (“Cerradão”), and vast portions with grasses. The DA has 19.5% of its area remaining from preserved forest, located within the limits of the studied area. Of the remaining 80.5%, 15.5% have grass vegetation and 65% have exposed soil; 76.7% of this is composed of the remaining B horizon and 23.3% of the C horizon. In the study region, the soil originally found was a red dystrophic oxisol, derived from migmatitic orthogneisses [25]. The DA soil had a Bw horizon with a clay texture and weak to moderate granular structure and an A horizon with a dark red color and thickness between 10–20 cm [26]. With the degradation process caused by the construction of the dam, the DA currently presents, in most of its extension, thin remaining layers of the B horizon and, locally, the C horizon is exposed, this one with sandy texture and whitish coloring [26]. The region is located in the Cerrado biome, and has vegetation characteristic of transition from Cerrado to Atlantic Forest [27]. As in a large part of the Cerrado, the DA environment suffered intense fragmentation of this biome due to the intense agro-pastoral activity [28]. Although the area is owned by Companhia Energética de Minas Gerais (CEMIG) (Energy Company of Minas Gerais), private properties are found in its surroundings where there is agricultural activity, especially downstream from the DA.

**Fig 1:**
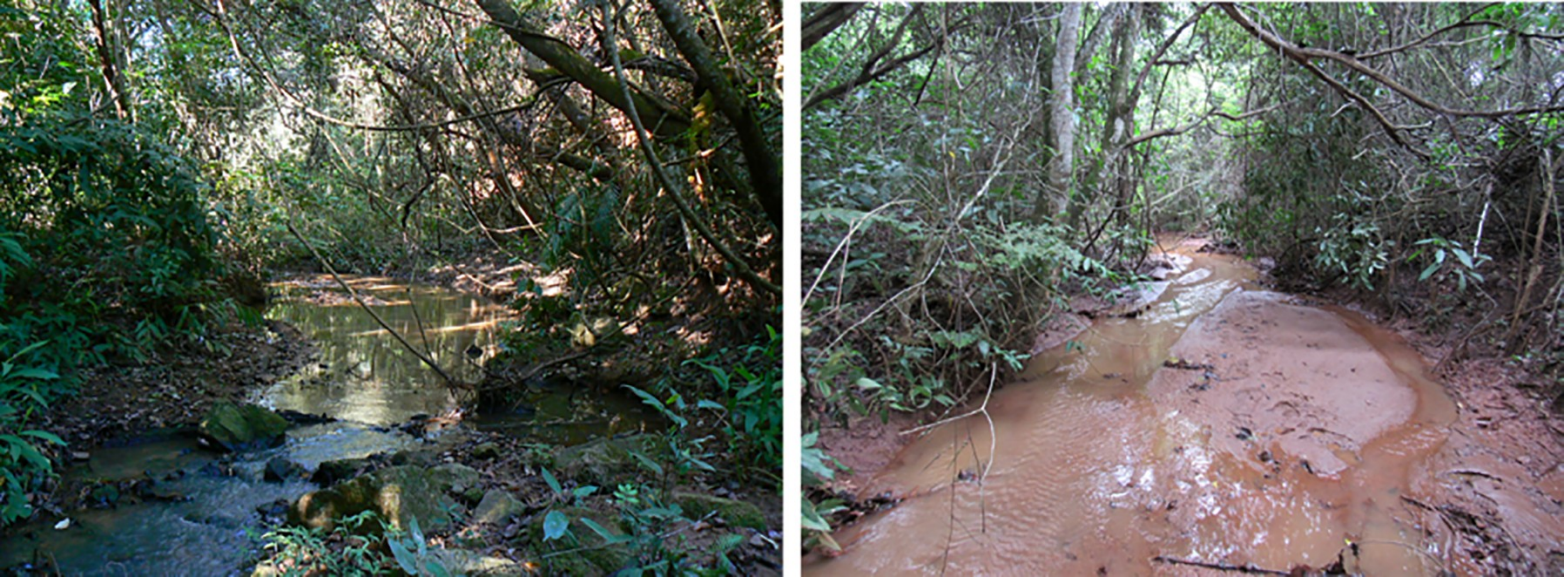
Images of stretches of streams studied in Catalão, GO, Brazil. (a) Note the presence of the preserved riparian forest and the absence of evidence of silting in the current reference (RS). (b) The silting state of the degraded stream (DS). Photos: Cláudio Tavares-Jr. (Personal archive).

During a rehabilitation project carried out in the DA between 2001 and 2002, structures were built to collect and control rainwater drainage. The source and part of the bed of the Pedra Branca stream (degraded stream—DS), which was found partially silted (Fig 1), are also located within the area. It is in this stream that most of the material drained by the aforementioned drainage network flows. About 5 km from the DA is the Olhos d’Agua stream, used as a reference stream (RS), which was not directly influenced by the impacts caused by the construction of the Emborcação HPP. The RS is located in a matrix of native forest, with the presence of well-structured riparian forest and also with rural properties in its surroundings with intense agro-pastoral activity (Fig 1). Both the DS and the RS are second-order streams [29], tributaries of the Paranaíba River basin.

The region’s climate is predominantly Aw [30], with dry winters [31]. Data from the Catalão weather station (INMET station: 83526) show that the average annual rainfall in the last 20 years was 119.83 mm, with the rainiest year being 2017, with an average of 155.36 mm. The region had an average of 118 rainy days per year for the past 20 years, two days with accumulated daily rainfall above 100 mm/day and 77 days above 50 mm/day. During the monitored period, between April-2019 to April-2020, there were 160 rainy days, making a total of 1648 mm of accumulated rainfall, with a torrential rain event (153 mm) and eight days above 50 mm/day. The average temperature in the region is 23° C, with 84 days with maximum temperature above 30° C in the last 20 years [32].

### Digital cartography

For the creation of hypsometric and use and occupation maps of the sub-basins of the studied streams, there were used images that were obtained by the sensors on board the satellites Sentinel-2 and ALOS (Advanced Land Observing Satellite), with spatial resolution of 10m and 12.5m, respectively. The images captured by Sentinel-2 provided the mapping of land use and facilitated the detailing of the physiognomy of rivers in the study area. The image captured by the PALSAR sensor (Phased Array L-band Synthetic Aperture Radar), on board ALOS, allowed to delimit the areas of the hydrographic sub-basins, as well as to develop a digital elevation model (DEM). The maps were made using the ArcGis 10.3 software.

### Field data collection

Three field campaigns were carried out over a hydrological year, in order to characterize the beginning of the dry season (May/19), the end of the dry season (September/19) and the end of the rainy season (March/20). Five points were defined along a stretch of 600 m in each stream (RS and DS) for river characterization and sampling. The length of the stretch was defined by the area where the stream was subjected only to the impact caused by the DA, that is, before the stream entered private rural properties, where it would be subject to other impacts, such as damming and grazing cattle. For the characterization, data on the physical and chemical conditions of the environments were obtained, including the liquid flow (m^3^/s), the slope of the RS and DS channels (m) and the granulometric and geochemical composition of the sediments.

To calculate the average flow of the channels in the cross sections, the instantaneous speed (m/s) was measured using a flow probe (Flowatch^®^) and the depth (m) measured with a level ruler. The measurements were made according to the width of the sampled section, ranging from 3–7 measurement points in the transversal direction per sampling point [33,34]. To obtain the average speed in the section (m^3^/s), the number of instantaneous speed measurements varied according to the depths found. Thus, following the methodology proposed by [35], at depths less than 10 cm, the average speed was considered as the instantaneous speed measured at only one depth (0.6 cm from the surface). At depths greater than 10 cm, speed was measured at two depths (0.2 cm from the surface and 0.8 cm from the bottom), with the assumed average speed being equal to the arithmetic average between the two. The method used to determine the average flow rate in the cross section was the vertical slices integration method, where the cross section of a channel was divided into segments corresponding to the number of vertical slices defined in the field. The average speed in each vertical slice was then multiplied by the corresponding area. Thus, the sum of the partial flows of each vertical slice corresponds to the total average flow of the channel in the analyzed cross section [35].

The chosen points had their cross sections mapped with the aid of an automatic level (CST/BERGER-SAL32X), measuring tape and a crosshair. Boards were built in the cross sections of the channels based on the slope data, for the monitoring of the seasonal variations. For the characterization of the contribution of the DA soil in the constitution of the DS sediment, soil samples from the DA area, from the bottom sediment of the channel bed and of the outcropping soil were collected. Sediment samples from the channel bed and soil from the banks were obtained every 50 m in the studied streams, totaling nine samples per stream. The DA soil was sampled in 23 equidistant points, distant about 300 m from the riverbed of the DS.

The samples of the sediment and soil (300g) were removed (first 20 cm depth) with the aid of a plastic shovel. In laboratory, the samples were dried in an oven at 40° C [36], and analyzed for for physical (granulometry) and geochemical features at the Laboratory of Soil Analysis, Vegetable Tissue and Fertilizer of the Department of Soils at the Federal University of Viçosa. In the granulometric analysis, dry sieving and pipetting methods were used [36]. We individualized fractions of coarse sand (2 to 0.2 mm); fine sand (0.2 to 0.05 mm), silt (0.05 to 0.002 mm) and clay (<0.002 mm). In the geochemical analysis, the levels of phosphorus (P) and potassium (K) were benchmarked by Mehlich -1 Extractor; the calcium ions (Ca^2+^) and magnesium (Mg^2+^) and exchangeable aluminum (Al^3^^+^), were benchmarked by Extractor: KCl -1 mol/L; potential acidity (H + Al) were benchmarked by Calcium Acetate Extractor 0.5 mol/L—pH 7.0. The exchangeable bases (SB) and organic matter (OM) were determined through C.Org x 1.724 -Walkley-Black.

### Statistical analyzes

Generalized linear models (GLM) were constructed to test whether there was a difference between the flow of RS and DS. The GLMs had flow variables as explanatory variables and streams and seasonal seasons of the study period as explanatory variables. A generalized linear model (GLM) was also used to test whether there was a significant difference between the average particle size of the sediments and the soils of the two streams. The GLM had the particle size as a response variable of the sediment and the soil and the streams as an explanatory variable. To determine the difference between the categories, a contrast analysis was performed.

A principal component analysis (PCA) was carried out using the R “vegan” package [37], with data on the chemical composition and organic matter of the sediment samples to identify possible differences between the two streams. All analyzes were performed using the R software [38], with results with p <0.05 being considered significant.

## Results

**Fig 2:**
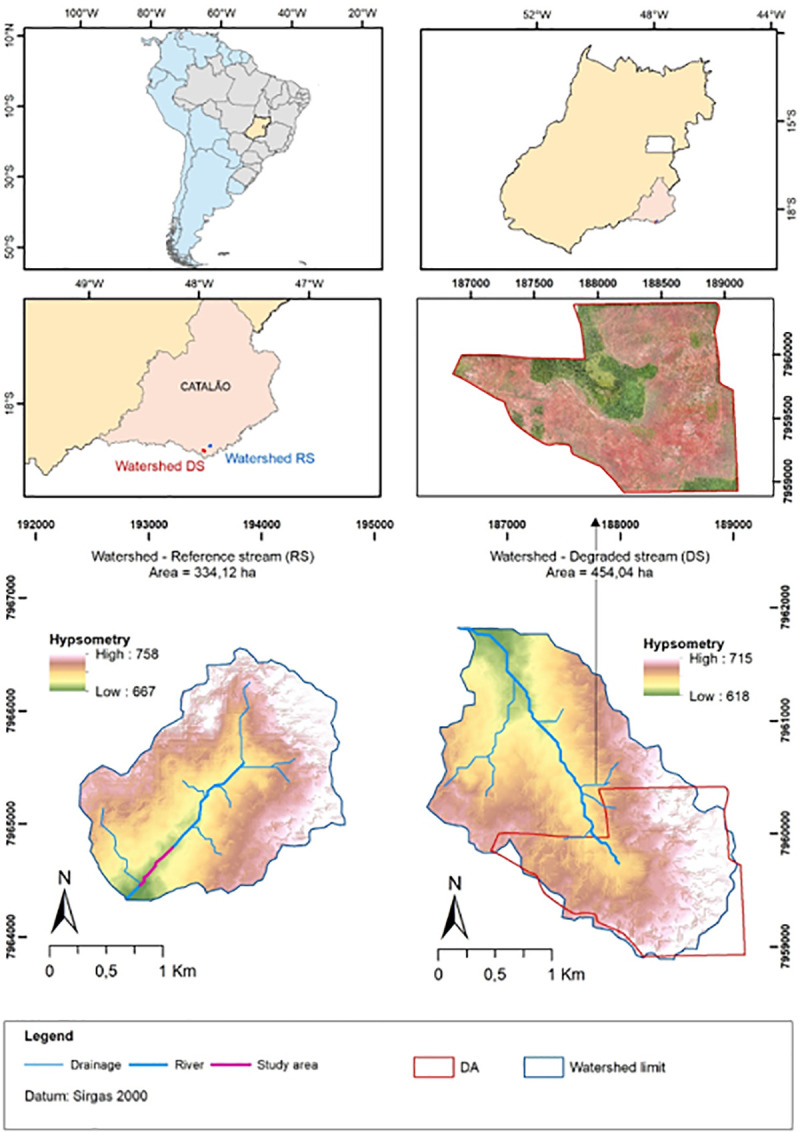
Degraded area (DA) location map. The figure also includes hypsometric maps of the sub-basins of the reference stream (RS) and the degraded stream (DS), including the values of their respective areas.

The Pedra Branca stream (degraded stream—DS) and the reference stream (RS) had similar regional geomorphological characteristics. The stretches had altimetry varying between 667 and 758 m (RS) and 618 and 715 m (DS), with average slopes of 9.1% (RS) and 9.7% (DS). The areas of its sub-basins are 334.12 m^2^ and 454.04 m^2^, respectively (Fig 2). Despite the region and the soils of their banks being similar, the two streams showed differences in their hydrological behavior and in the physical and chemical parameters of their sediments. Differences in chemical composition of the fluvial sediments from DS and RS were marked by the concentrations of K^+^ (135.56 mg/dm^3^ in RS and 44.55 in DS) and P (5.63 mg/dm^3^ in RS and 0.90 mg/dm^3^ in DS). Concentrations of CA^2+^ were higher in DS sediments, due liming process in the DA. Sediments from RS tends to be richer in phosphorous (5.63 mg/dm^3^average) in relation to DS (0.90 mg/dm^3^), while organic matter contents of the sediments were similar in RS (0.50 dag/Kg) and in DS (0.74 dag/Kg). Slightly finer-grained sediment was dominant in RS (average 0.70 mm mean particle size soil) in relation to DS (0.48 mm mean particle size soil) (Table 1).

**Table 1:** Physical and chemical parameters (mean ± standard deviation) of the sediment of the reference stream (RS) and the degraded stream (DS) in Catalão, GO, Brazil.

Parameters	Reference stream	Degraded stream
Al^3+^ (cmol_c_/dm^3^)	0.02 ±0.06	0.02 ±0.06
Ca^2+^ (cmol_c_/dm^3^)	0.80 ±0.24	1.46 ±0.55
H+Al (cmol_c_/dm^3^)	0.98 ±0.44	1.27 ±0.59
K (mg/dm^3^)	13.56 ±6.31	44.33 ±9.32
Mg^2+^ (cmol_c_/dm^3^)	0.26 ±0.07	0.60 ±0.24
P (mg/dm^3^)	5.63 ±1.18	0.90 ±0.51
OM (dag/Kg)	0.50 ±0.88	0.74 ±0.42
SB (cmol_c_/dm^3^)	1.09 ±0.32	2.17 ±0.80
Mean particle size sediment (mm)	1.41 ±0.08	0.56 ±0.06
Mean particle size soil (mm)	0.70 ±0.16	0.48 ±0.2
Flow (m^3^/s)	0.0088 ±0.009	0.0089 ±0.008

Al^3+^ = exchangeable aluminum, Ca^2+^ = calcium ions, H.Al = potential acidity, K = potassium, Mg^2+^ = magnesium ions, OM = organic matter, P = phosphorus and SB = sum of exchangeable bases.

**Fig 3:**
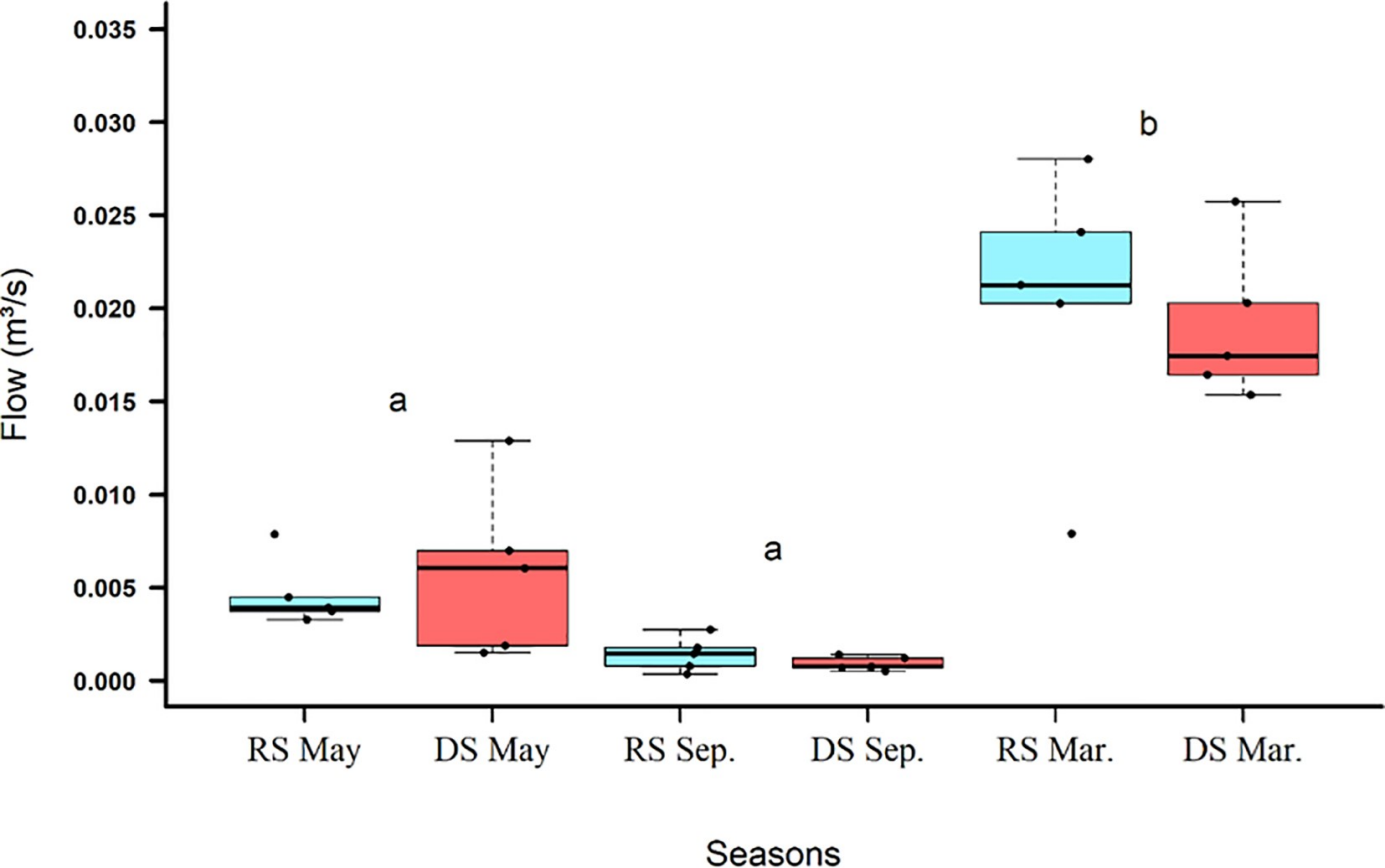
Box-plot graphs with data from flow (m^3^/s) over a hydrological year (May/19, September/19 and March/20) of the reference stream (RS) and the degraded stream (DS) in Catalão, GO, Brazil.

There was no significant difference between the flows measured in RS and DS (Table 2). However, the net discharge showed a significant difference in relation to the seasonal periods (Table 2). The flow (m^3^/s) followed the precipitation patterns registered in the Catalão rainy season, being higher at the end of the rainy season (March/2020; Figs 5 and 6). The RS flow rate was higher than the DS flow in all seasons except during the beginning of the drought (May/2019; Fig 3).

**Table 2:** Models of tests created for the variables of the reference stream (RS) and the degraded stream (DS) in Catalão, GO, Brazil.

Response variables	Explanatory variables	Family	F-Value	Df	Deviance	p-Value
Particle size	Sites	Quasibinomial	20.395	35	6.1624	<0.001*
Flow	Sites	Gaussian	0.0033	29	0.0009	0.9544
Flow	Seasons	Gaussian	22.737	29	0.0018	<0.001*

* statistically significant values.

**Fig 4:**
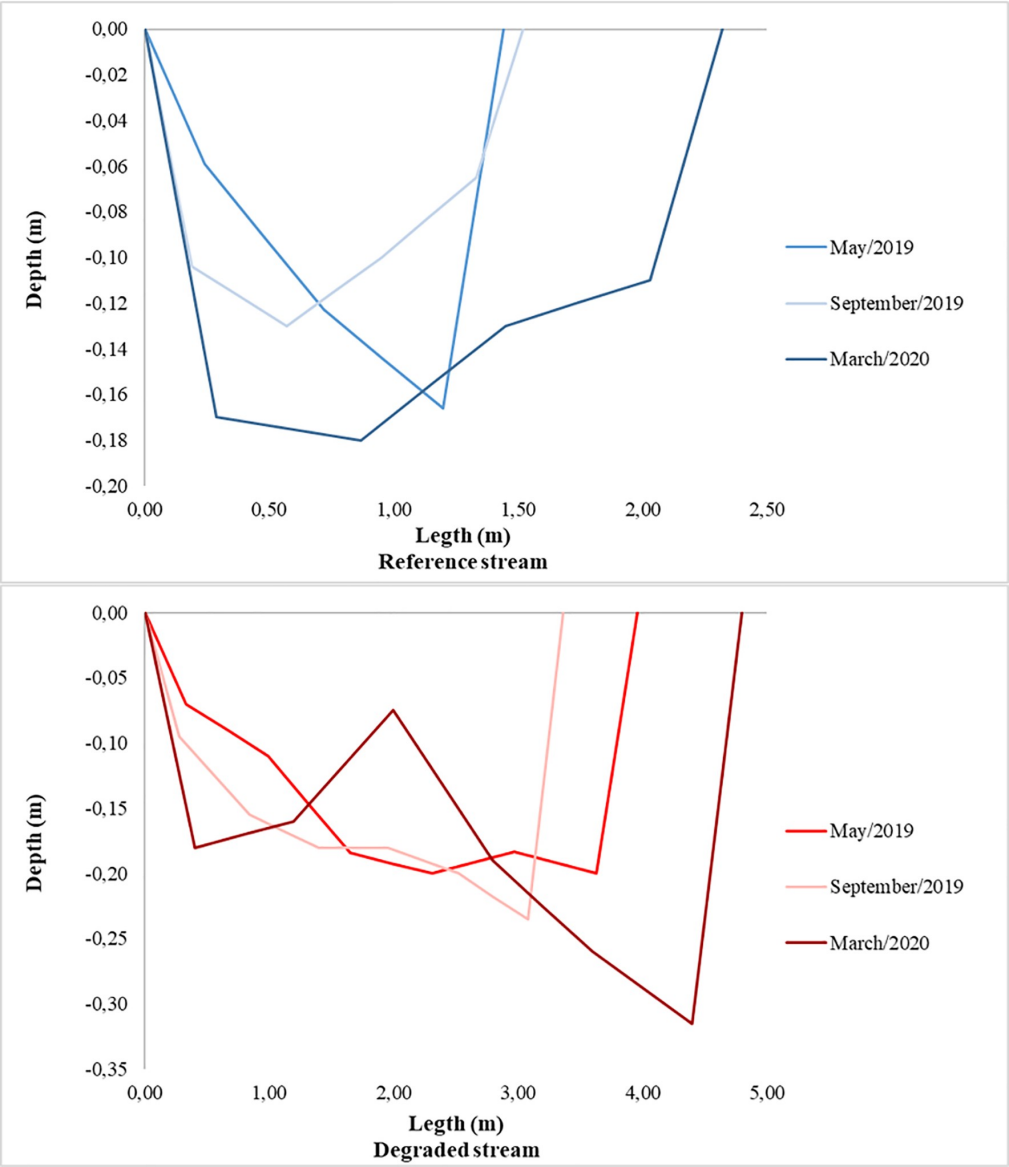
Profiles of the cross-sections further downstream of the reference stream (RS) and the degraded stream (DS) in Catalão, GO, Brazil. The profiles correspond to the beginning of the dry season (May/2019), end of the dry season (September/2019) and end of the subsequent rainy season (March/2020) on the same sites in the RS e DS.

Both streams are relatively shallow, with their average water depths increasing from upstream to downstream and at the end of the rainy season, with the lowest water volumes at the end of the dry season. Despite this, cross-sectional profiles show different responses to seasonal changes. The RS presented a greater stability of its transversal profiles, when compared with the variations observed in the DS channel. The formation of sediment bars in the DS can be clearly seen in several of its transversal profiles (Figs 1 and 4). These differences are more easily observed in the sections further downstream of the two monitored stretches, especially in the profile performed at the end of the rainy season (March/2020; Fig 4).

**Fig 5:**
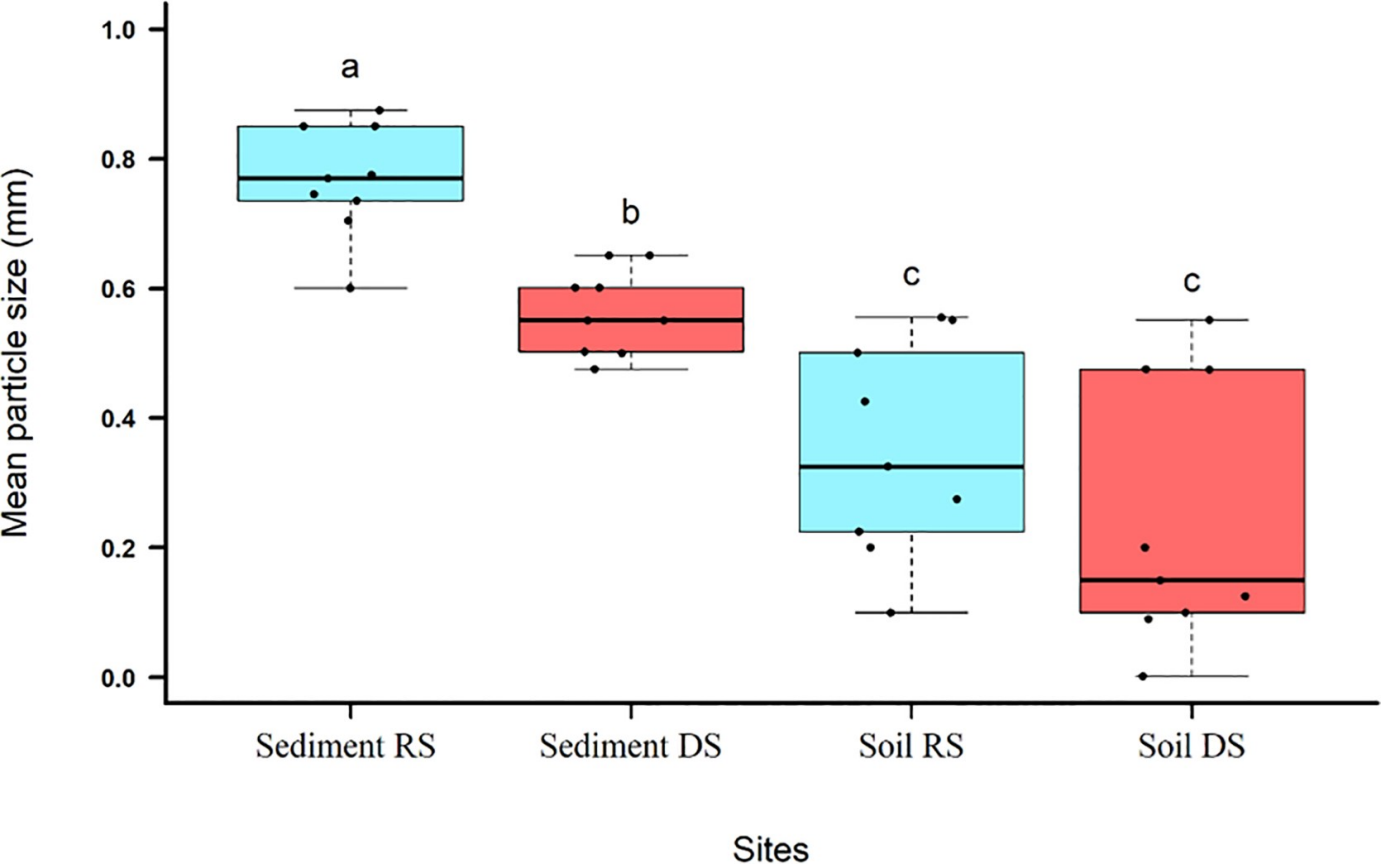
Box-plot type graphics with data from the granulometry of the sediment and soil of the banks of the reference stream (RS) and the degraded stream (DS) in Catalão, GO, Brazil. p-value <0.001.

The sediments of the beds of RS and DS streams show a significant difference in relation to the average size of the particles (Table 2; Fig 5). The RS presents thicker sediments (coarse sands and granules), while in the DS the fine to medium sands are predominant (Fig 5). In addition, the granulometry of the bed sediments differs significantly from that of the soils on their respective banks (Fig 5) and is composed of thicker material. The soils on the banks of the two streams did not show any significant difference in relation to the average size of their particles (Fig 5).

**Fig 6:**
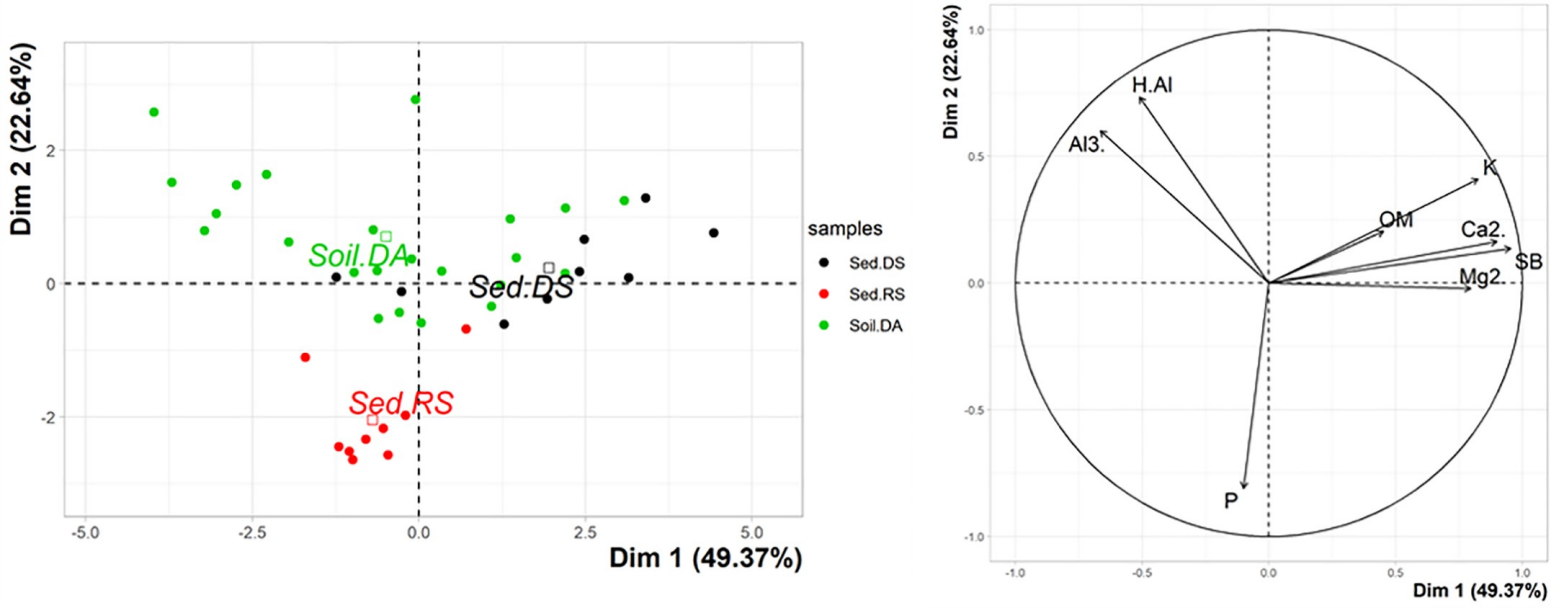
Analysis of main components of the sediment of the reference stream (RS) and degraded stream (DS) and soil of the degraded area (DA) in Catalão, GO, Brazil. Subtitle. Al3.-Exchangeable aluminum, Ca2.-Calcium ions, H.Al-Potential acidity, K-Potassium, Mg2.-Magnesium ions, OM-Organic matter, P-Phosphorus and SB-Sum of exchangeable bases.

The principal component analysis (PCA) in relation to the chemical composition of the sediments of the streams and the soil of the degraded area (DA), showed a clear separation between the RS and DS sediments and a similarity between the DA soil and the sediment of DS. The first axis explains 49.37% of the variation, while the second axis explains 22.64%. RS presents a greater contribution of the element phosphorus (P) in its sediment and a negative relationship with potassium (K) and magnesium (Mg^2+^) and calcium ions (Ca^2+^; Fig 6). The DS sediment and the DA soil have a low contribution of organic matter (OM) and a positive relationship with potassium (K) and magnesium (Mg^2+^) and calcium ions (Ca^2+^). The DA soil still has a positive relationship with exchangeable aluminum (Al^3+^) and potential acidity (H + Al; Fig 6).

## Discussion

The similar geomorphological characteristics of the two streams analyzed (geology, altimetry, rainfall, area of ​​the sub-basins) cause a similar behavior of the net flow during the seasonal seasons. The great discrepancy between the two streams is in the use and occupation of their sub-basins. Despite presenting narrow riparian forests and both being inserted in an agro-pastoral region, the Pedra Branca (DS) stream has most of its headland located in the DA. This area, despite having been submitted to a restoration project, is deforested and with exposed soil (predominantly horizon C). As a consequence, the soil compaction in the area is much higher than the adjacent native forest areas [26], favoring runoff over infiltration [39]. This is because, in addition to being a physical barrier to runoff, reducing its speed, vegetation also promotes greater macroporosity to the soil, which favors infiltration [40,41].

There is an intimate connection between lotic environments and rainfall [42–44]. The network of channels and, mainly, the flow that form the lotic environments can present expansion or retraction according to the seasonal rains or individual events, as in torrential rains [18]. Both streams showed higher flows during the rainy season and lower flows at the end of the drought, reflecting increased precipitation and aquifer recharge [20]. Only at the beginning of the drought did the DS show, in some points, a greater flow than RS, in the other stations the measurements of the net discharge of RS were always higher. The largest variances in the DS flow data both at the beginning of the dry season and at the end of the rainy season are a reflection of precipitation in these periods. Due to the deforestation of DA the precipitation turns into surface runoff, captured by the drains, being directed to the DS. In contrast, the preserved recharge area of ​​RS favors infiltration, allowing a more effective recharge of the aquifer, which maintains its base flow during the drought and increases its net discharge at the end of the rainy season [20].

Different hydrogeomorphological conditions were found in the reference (RS) and degraded (DS) streams. RS is in a more preserved condition, with high heterogeneity in the substrate of its bed. The DS is silted up over the entire length of the studied section, with a high level of substrate homogenization and with sediment bars, whose system energy during the rainy season was unable to remove [45,46]. These characteristics are probably a reflection of the transport of a large load of sediments from the degraded area upstream [7,47]. In DA, runoff is markedly affected both by the absence of vegetation and by the characteristics of high rainfall intensity, which occur mainly between October and March. In hydrographic basins where vegetation cover has been removed, the action of intense rain increases the occurrence and magnitude of soil loss [15,48]. Under these conditions, rainfall events enhance degradation processes, trigger erosive processes, increase sediment transport and, consequently, promote changes in the hydro-sedimentological balance of water courses downstream of these areas, culminating in the silting up of rivers and streams [44,49,50].

It is interesting to note that the mean diameter of the sediment was significantly different between RS and DS. The DS presents much finer particles than those present in RS, with sizes similar to the remaining soil found in the DA by [26]. This condition may be a reflection of the sedimentary load resulting from erosive processes and the channeling of laminar flows that occurs in the degraded area at its head [39,51]. This sedimentary contribution also influences the chemical compositions of the sediments, promoting a greater concentration of bases in the DS [52,53]. These differences point to areas of sediment upstream from the monitored stretches [22], since the main sources of sediment for the lotic systems are the bed material, the erosion of the banks and the sediments upstream [51]. The similarity between the chemical composition between the DA soil and the DS sediment, as well as the absence of fine particles in RS sediments corroborates the hypothesis of sediment input from the DA. Therefore, although the soils on both banks of the stream are similar in terms of particle size, with no difference between each other, both differ significantly from the sediments.

Rivers and small streams are among the natural resources that are most affected by environmental degradation processes [7,54]. Many lotic systems, such as the Pedra Branca stream, have impacts caused by anthropic changes along their hydrographic basins, resulting in changes in the flow regime, silting and, consequently, habitat degradation with biodiversity loss [6,49,55,56]. The magnitude of environmental impacts related to the construction of large hydroelectric plants often overshadows secondary impacts related to their construction. This is undoubtedly the case for impacts related to the borrow pits [12,57]. The present study showed that degraded borrow pits can have a great influence on the hydro-sedimentary imbalance of watercourses under its influence. The suppression of vegetation combined with the removal of the superficial layers of the soil changed the water cycle, reducing the recharge of aquifers and increasing the runoff. This increase in laminar flow intensified the erosive processes. Channeled flows led sediments to the Pedra Branca stream, changing its metastable dynamic balance and promoting its silting, with the formation of bars inside the channel, whose physical and chemical characteristics reflect those of the degraded area for the construction of the dam. The results show the need to review the intervention protocols in borrow pits and the environmental legislation that regulates their rehabilitation.

To mitigate the scenario found in the Pedra Branca stream, it would be extremely urgent the restauration of the forest cover of the borrow pit area. With the restructuring of the vegetation cover and resulting restructuring of the soil, the erosion processes and the contribution of sediments to the stream bed would decrease. Another important short-term action would be the installation of sediment collectors in the drainage networks as well as their maintenance. Much of the rainwater is dumped into the Pedra Branca stream, as well as all the sediment transported by them. It would be necessary that these structures could trap part of the sediment transported avoiding the critical sedimentation of the Pedra Branca streambed.

## Supporting information

S1 Data (XLSX)
